# Interaction of the Zika virus with the cytoplasmic dynein-1

**DOI:** 10.1186/s12985-023-01992-6

**Published:** 2023-03-06

**Authors:** Dan Israel Zavala-Vargas, Giovani Visoso-Carbajal, Leticia Cedillo-Barrón, Jessica Georgina Filisola-Villaseñor, Romel Rosales-Ramirez, Juan E. Ludert, Edgar Morales-Ríos

**Affiliations:** 1 Department of Biochemistry, Center for Research and Advanced Studies (Cinvestav), 07360 Mexico City, Mexico; 2 Department of Molecular Biomedicine, Center for Research and Advanced Studies (Cinvestav), 07360 Mexico City, Mexico; 3 Department of Infectomics and Molecular Pathogenesis, Center for Research and Advanced Studies (Cinvestav), 07360 Mexico City, Mexico

**Keywords:** Zika, Zika virus, Envelope protein (E), Dynein, Virion transport

## Abstract

Zika virus (ZIKV) infection is a major public health threat, making the study of its biology a matter of great importance. By analyzing the viral–host protein interactions, new drug targets may be proposed. In this work, we showed that human cytoplasmic dynein-1 (Dyn) interacts with the envelope protein (E) of ZIKV. Biochemical evidence indicates that the E protein and the dimerization domain of the heavy chain of Dyn binds directly without dynactin or any cargo adaptor. Analysis of this interactions in infected Vero cells by proximity ligation assay suggest that the E–Dyn interaction is dynamic and finely tuned along the replication cycle. Altogether, our results suggest new steps in the replication cycle of the ZIKV for virion transport and indicate a suitable molecular target to modulate infection by ZIKV.

**Supplementary Information:**

The online version contains supplementary material available at 10.1186/s12985-023-01992-6.

## Introduction

Mosquito borne flaviviruses are a great public health burden in countries located in tropical and subtropical areas. Flaviviruses such as dengue virus (DENV) or yellow fever (YFV) cause systemic infections that in severe cases result in liver failure, hemorrhagic syndromes, and vascular compromise that, if not treated properly, can result in the death of the patient. Zika virus infections usually course with mild symptoms, but ZIKV infection during pregnancy can cause injury to the placenta and can be transmitted to the developing fetus, resulting in placental insufficiency, microcephaly, congenital malformations, and fetal demise [1]. In addition, ZIKV can also infect the human reproductive tracts leading to sexual transmission, and testis damage [2]. Currently, there is no specific antiviral treatment for ZIKV infected patients. Thus, a better understanding of the ZIKV-cell interactions, could lead to a better design for drugs that prevent or inhibits infection by this virus. ZIKV is taxonomically classified as a member of the Flaviviridae family, that affects mammals and birds [3]. The ZIKV replication cycle starts when a mature virus enters a susceptible cell via receptor mediated endocytosis, and the virion is internalized encapsulated in an endosome; there the E protein undergoes conformational changes when exposed to low pH, due to the endosome acidification by the V-ATPase [4]. The fusion of the viral envelope with the endosome membrane, mediated by the exposed fusion loops of the E protein, results in the release of the ZIKV 11 Kb single-stranded positive RNA genome, into the cytoplasm [5]. The RNA is translated as a single polyprotein in the endoplasmic reticulum (ER), where it undergoes proteolytic cleavages by either host and viral proteases to generate 10 different mature proteins; 3 structural proteins, the capsid C protein (C), the pr-M protein and the envelope E protein, and 7 non-structural proteins denominated NS1, NS2A, NS2B, NS3, NS4A, NS4B, and NS5 [6]. Since viruses are non-motile macromolecules, they hijack the host cytoskeleton molecular motors to move inside the eukaryotic cell and complete their replication cycle [6]. Human cytoplasmic dynein-1 (dynein) is a 1.5 MDa (1500 kDa) molecular motor that, together with its cofactor, dynactin, transports different cargoes to the minus end of the microtubules (MT) in an ATP dependent manner [7]. Dynein is a homodimer composed of 6 different proteins: the heavy chain (HC), which acts like a scaffold where the intermediate chain (IC) and the light intermediate chain (LIC), bind. The light chain 8 (LC8), light chain 7 (LC7 or roadblock) and the t-complex testis-specific protein 1 (Tctex1) bind to the IC [8]. The HC contains a AAA-motif, that hydrolyzes ATP to produce conformational changes in the microtubule-binding domain (MBD) for its binding–unbinding from the MT [9]. Dynein–dynactin complex recruits several cargo adaptors to cope with the transport of different cargoes such as single proteins or protein aggregates, endosomes, melanosomes, autophagosomes, lipid droplets, peroxisomes, mitochondria, endoplasmic reticulum, Golgi apparatus, and the vesicles de rived from these organelles [6]. Dynein also participates in the replication cycle of a great number of viruses from different families including the Flaviviridae family [10]. This interaction could be direct with the virion or indirect by inter- acting with virus containing vesicles. In any case, the result is the transport of the viral particles into the perinuclear area over the microtubules [6].

## Background

There is evidence that shows that ZIKV infection induces massive changes in the host’s cytoskeleton [11]. In this condition, the cytoskeleton produces a microtubule-dependent cage that surrounds the viral factories, and addition of paclitaxel, a drug that stabilizes microtubules against depolymerization, affects virus replication [11]. Moreover, evidence of direct interaction of dynein with DENV E protein have been obtained by immunoprecipitation [12]. In this work, we show data indicating that the ZIKV E protein binds directly to the dimerization domain of the HC of dynein in a non-vesicle nor dynactin fashion. These results strongly suggest that dynein participates in the ZIKV replication cycle and open a window for the development of new drugs for patient treatment.

## Results

### Naturally occurring dynein HC binds to the ZIKV in vivo.

#### Envelope-dynein HC colocalization assay

**Fig. 1:**
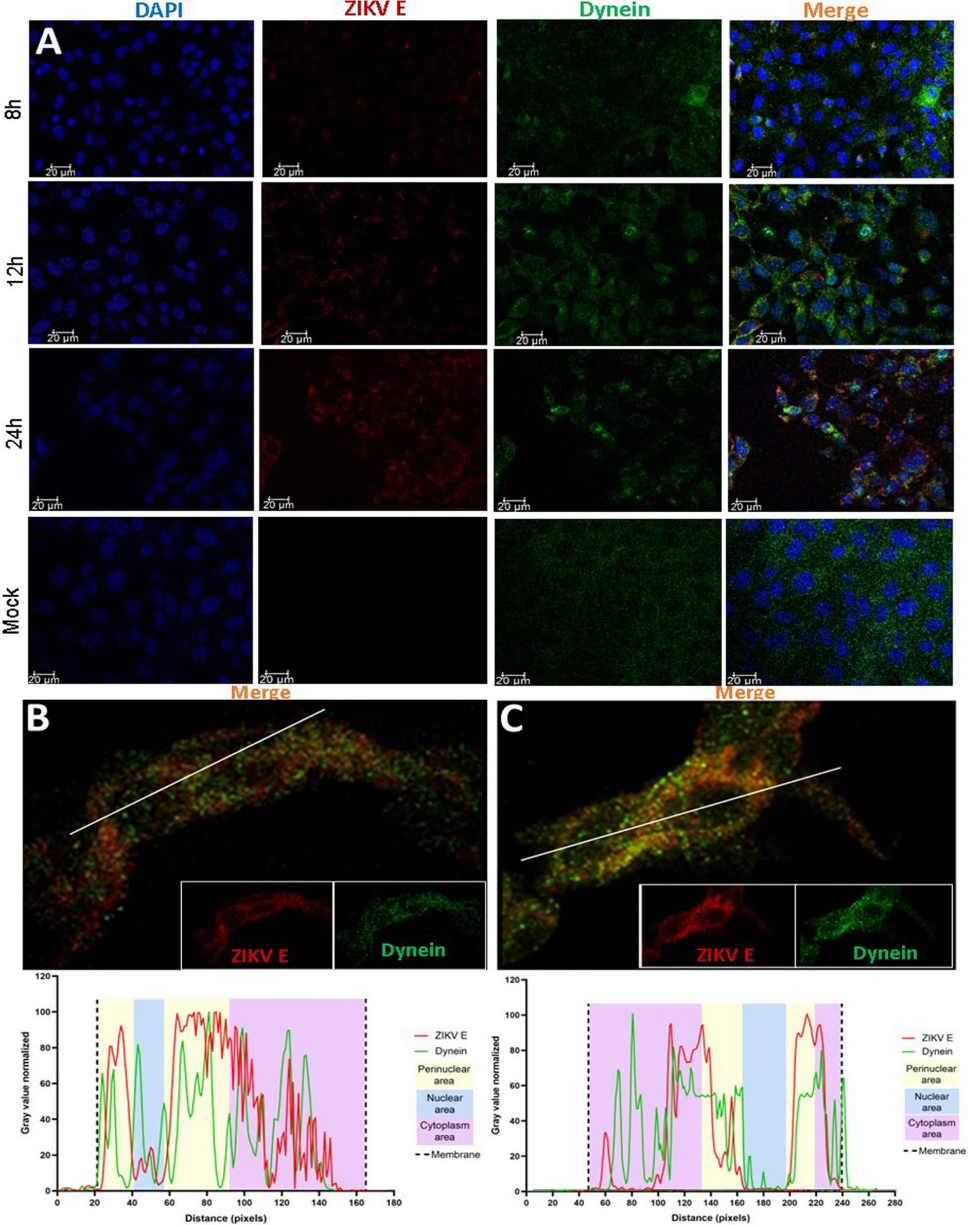
Immunofluorescence Assay of ZIKV infected cells. **A** Vero cells were infected with ZIKV and fixed at different times post-infection (p.i.). ZIKV pE was marked with mouse MAb and a secondary anti-mouse IgG Alexa 488 (red). Dynein HC was marked with rabbit polyclonal Ab and an anti-rabbit IgG Alexa 594 (green). Nuclei were stained with DAPI (blue). Split images showing the colocalization in a 12, 24, and 48 h time course. Mock cells are cells without infection fixed at 48 h. **B** Magnified immunofluorescence image at 12 h post-infection. **C** Magnified immunofluorescence image at 24 h post-infection.The lower panel of figure **B** and **C** shows the colocalization profiles of the sections marked with the white line. The green line is Dynein-HC and the red line is pE from the ZIKV. The colored areas are: Light yellow perinuclear section, blue is the nuclear area, magenta is the cytoplasm area, and the dotted line is the plasma membrane

In Fig. 1A we show the ZIKV envelope protein–dynein HC colocalization pattern at 8 h, 12 h, and 24 h. Dynein HC staining in non-infected cells was uniformly distributed, whereas in infected cells, clear zones of greater intensity are seen around the nuclei, even in the cells in which the envelope protein is poorly visible. Throughout the experiment, we have shown a notable increase of dynein HC fluorescence around E protein fluorescence. At 8 h, E protein is shown in low quantity. As time progressed, the amount of E protein increased and at 12 h (Fig. 1B), colocalization zones distributed in the cytoplasm were observed with a Pearson’s colocalization index of 0.672. The colocalization distributions starts to accumulate at the perinuclear zone, which is more evident at 24 h. At 24 h (Fig. 1C), the Pearson’s colocalization index is 0.698 (Figure C, lower panel). This coincides with the synthesis and assembly of viral proteins near the ER [13].

#### Envelope-dynein HC proximity ligation assay

**Fig. 2:**
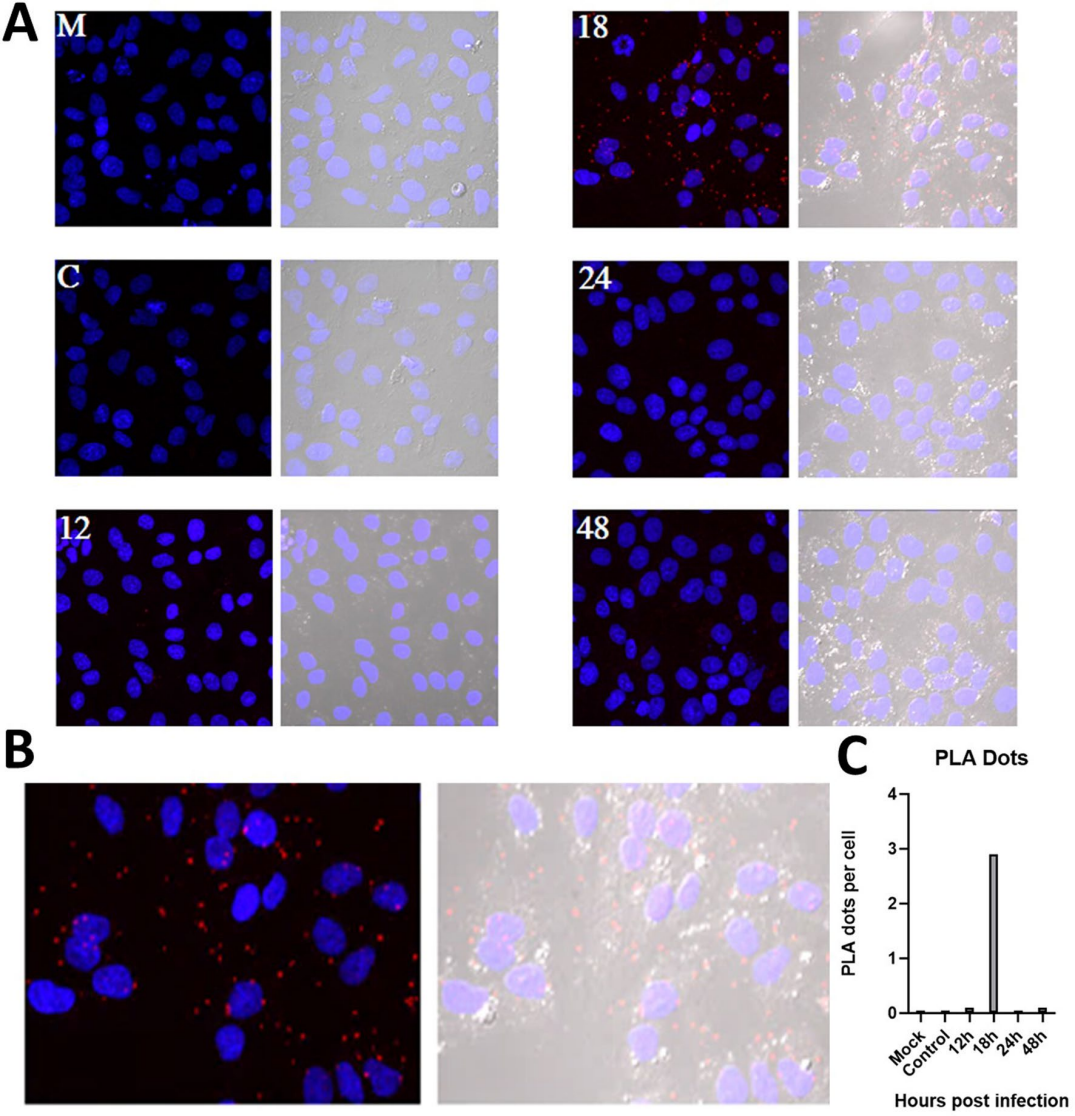
Proximity ligation assay of ZIKV infected cells. **A** (M) Mock image is cells without infection fixed at 48 h **C** control was cells infected with the virus at 18 h without the incubation of the anti-pE antibody. For the time course, we used Vero cell infected with ZIKV and fixed at different times post-infection: 12, 18, 24, and 48 h This assay was performed with a monoclonal anti-pE in muse MAb and with a polyclonal anti- NTDyn in rabbit. The PLA dots were marked with Cy3 (red). The images on the right show DAPI (blue) and Cy3 (red) channels. The images on the left show blue, red and the differential interference contrast channel (grey). **B** Magnified image at 18 h post-infection. C. PLA dots per cell graphic, from 100 cells. Show the highest value at 18 h.p.i. mean 2.9 PLA dots per cell

With the proximity ligation assay (PLA), we are evaluating the interaction of proteins at distances < 40 nm in ZIKV infected cells at 12, 18, 24 and 48 h.p.i. (Fig. 2). Weak positive signals (1 or fewer PLA dots per cell) for dynein HC-E protein interaction were observed at 12 and 24 h.p.i. but clear positive signals (6 or more dots per cell) were observed in cells fixed at 18 h.p.i. The absence of any signal in the 2 negative controls used corroborated the specificity of the assay. These results agree with the colocalization and protein expression results, which suggest that in ZIKV infected cells, dynein and the E protein interact in a narrow time window.

#### Overexpression of dynein HC in ZIKV infected cells

**Fig. 3:**
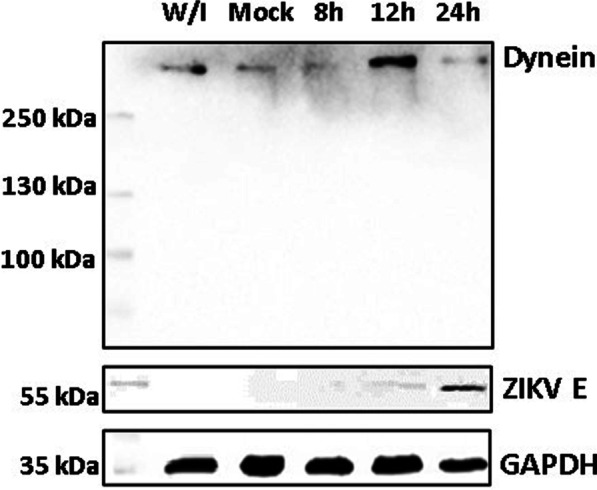
Immunoblot depicting ZIKV envelope protein and dynein HC expression kinetics. Lane 1. MW marker. Lane 2. Vero cells without infection. Lane 3. Mock infected, Vero cells infected with inactivated ZIKV. Lane 4. Harvested Vero cells 8 h.p.i. Lane 5. Harvested Vero cells 12 h.p.i. Lane 6. Harvested 24 h.p.i

We analyzed the expression of pE and dynein HC, during infection (Fig. 3), at 8, 12 and 24 h post infection by means of a WB. At 8 h, the envelope protein is undetectable, while dynein HC is observed with low intensity, but at 12 h, the envelope begins to be detectable while dynein HC presents a higher signal intensity. Finally, at 24 h the envelope protein increases its signal, but dynein HC seems to decrease, although the stoichiometric relationship does not seem to be equal, the kinetics is consistent with what was observed in colocalization. The results suggest that, at an early stage of the infection, dynein HC would have the function of transporting ZIKV in direct interaction with the E protein, for which it would necessarily be overexpressed. However, when this stage is finished, dynein HC expression goes back to the non-infected levels (Fig. 3 24h lane).

#### Dynein HC immunoprecipitation

**Fig. 4:**
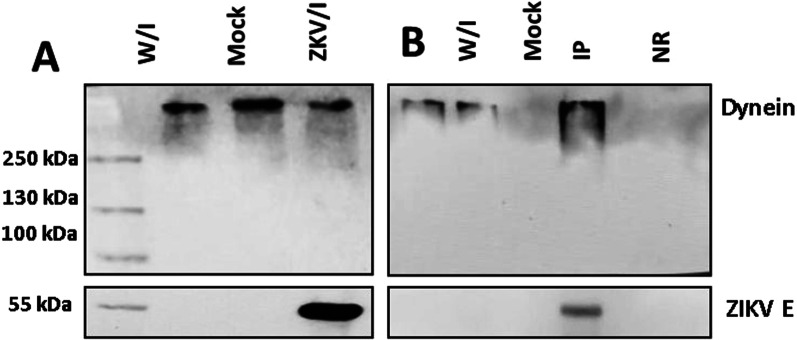
Immunoblot depicting immunoprecipitation Envelope protein–Dynein HC complex. **A** Control Western Blot. Lysates of ZIKV uninfected or uninfected Vero cells were analyzed with anti-E monoclonal antibody and anti-Dynein HC antibody followed by goat anti-rabbit HRP. Lane 1 MW marker, lane 2 uninfected Vero cells lysate, lane 3 Mock infected Vero cells lysate, lane 4 Zika infected Vero cells lysate. **B** Immunoprecipitation assay with anti-Dynein HC antibody, Lane 1 immunoprecipitation uninfected Vero cells lysate, Lane 2 immunoprecipitation mock infected (heat-inactivated virus) Vero cells lysate, Lane 3 immunoprecipitation ZIKV infected Vero cells lysate, Lane 4 immunoprecipitation with Anti-NDP52 antibody (no related) ZIKV infected Vero cells lysate

Since dynein HC is overexpressed in ZIKV-infected cells at 18–24 h, we captured it with its interactions during infection. First, we analyzed a control of infected cells that were lysed and where we observed the presence of both proteins, using WB and identified by a monoclonal mouse antibody directed against the dynein HC mAb and anti-E protein 4G2 mAb (Fig. 4A). Subsequently, we observed that the E protein of ZIKV appeared in the particulate fraction when dynein was immunoprecipitated using anti-HC antibodies together with coated sepharose A beads (Fig. 4B). There was no signal in the immunoprecipitated uninfected cells lysate in Lane 4. These results validated the colocalization and PLA of Envelope protein and dynein HC showing the time-dependent complex formation in vivo.

#### Zika virions interact with NTDyn

**Fig. 5:**
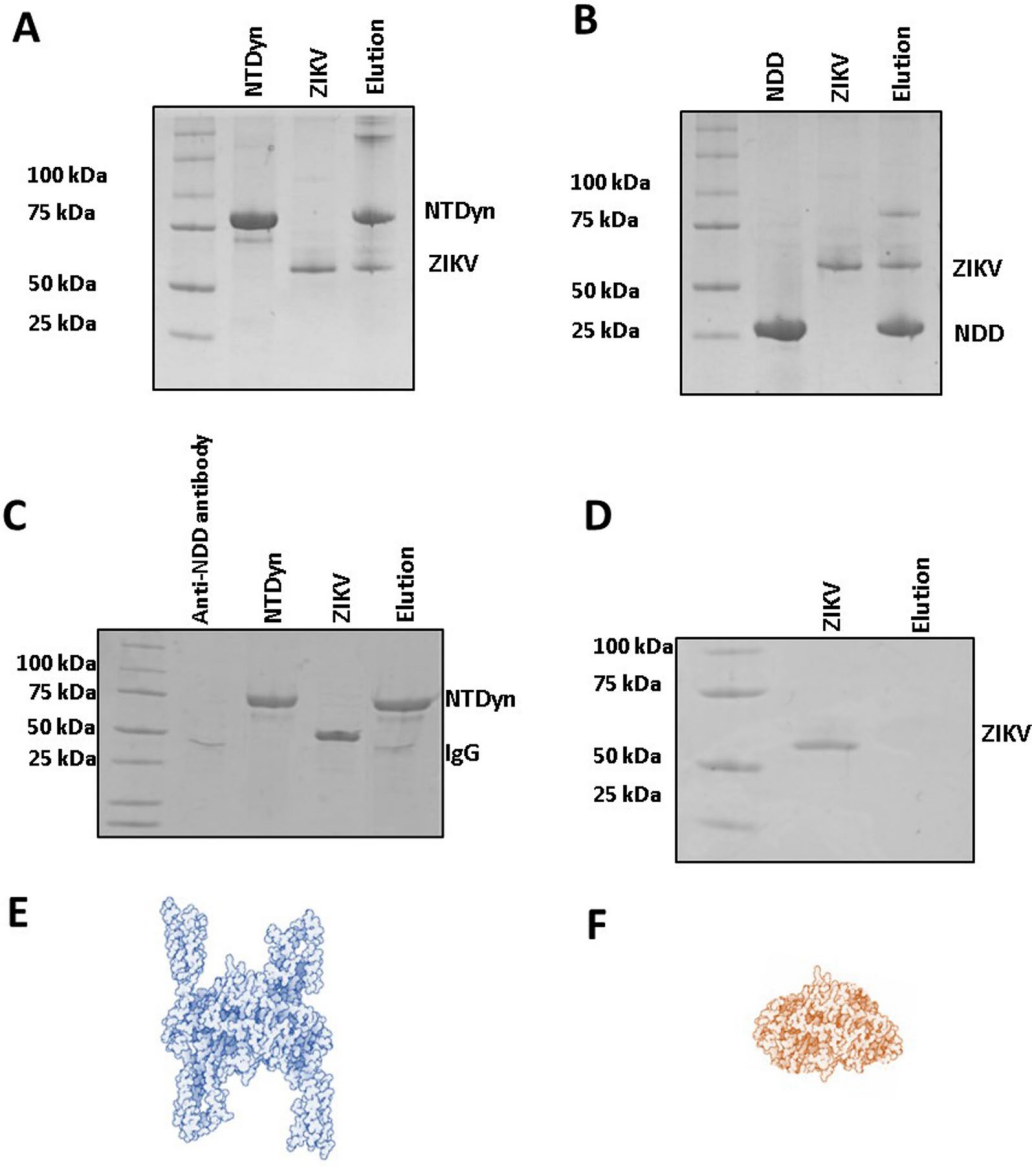
Coomassie-stained PAGE-SDS gel showing binding of the ZIKV with NTDyn. Purified ZIKV from Vero cells was loaded into a 5 ml HisTrap HP Nickel (GE) column pre-loaded with the His6NTDyn. The column was washed, and the sample was eluted with imidazole. The fractions were analyzed by SDS-PAGE. Lane 1. MW marker. Lane 2. The purified His6NTDyn band. Lane 3. Enriched pE from the ZIKV. Lane 4. The eluted fraction contains both, the complete ZIKV and NTDyn-His. Lane 1. MW marker. Lane 2. His6NDD purified protein bound to affinity column, Lane 3. Purified ZIKV. Lane 4. Elution, C. Lane 1. MW marker. Lane 2. His6NTDyn + anti-NDD antibody complex. Lane 3. Purified ZIKV. Lane 4. Elution. D. Lane 1. MW marker. Lane 2. Purified ZIKV. Lane 3. Elution. E. left panel, the structure of the blue NTDyn is shown, composed in its nucleus by the NDD and in its external part by 6 lateral alpha-helices. Modified images of 5NW4 [14] and 5OWO [15]. F. NDD. Dimerization domain of dynein in red color. Modified images of 5OWO

Now that we have detected and captured the interaction dynein-ZIKV in vivo, we evaluated whether the isolated ZIKV is interacting with the NTDyn (dynein Heavy Chain 1–571) recombinant protein. Since we have preliminary data suggesting that recombinant NTDyn and protein E from ZIKV interacts (Additional file 1, Fig 7), we bound the His6NTDyn into a His Trap Nickel column and then we loaded in the column an enriched sample of ZIKV, harvested from infected Vero cells. We washed the column, eluted the bound material with imidazole and analyzed the fractions by SDS-PAGE (Fig. 5A); lane 1 MW marker, lane 2 the purified His6NTDyn, in lane 3 the purified ZIKV, lane 4 eluted material where we can see the band corresponding to His6NTDyn and bands corresponding to the MW of the E protein we do not detect any other protein component of the ZIKV perhaps due to the chromatography, since we use 0.5 of imidazole for the elution, the virions are probably disassembling. With the idea of delimiting the interaction region, we injected the recombinant His6NDD (Fig. 5B), this is the central core of the His6NTDyn fragment (NDD structure Fig. 5F). In lane 1 MWM, lane 2 is the purified His6NDD, lane 3 purified ZIKV and lane 4 elution shows the presence of both bands, this result is consistent with the previous experiment, and it delimits the zone of interaction between ZIKV and dynein, this is in the dimerization domain. As a negative control, we used a lab-made polyclonal anti-NDD antibody (Additional file 1) to block this domain in the His6NTDyn fragment, to expose only the helical bundles 1, 2 and 3 of dynein’s HC, this complex was injected into an affinity column and subsequently washed and injected with the ZIKV particle and eluted. In Fig. 5C we observe in lane 1 MW marker, lane 2 NTDyn and anti-NDD antibody (IgG) bands, lane 3 band corresponding to the ZIKV envelope protein in lane 4 elution where we only observe the bands corresponding to the His6NTDyn-Anti NDD complex, the absence of bands corresponding to the envelope, shows no ZIKA-complex interaction, this result shows the specificity of the ZIKV–NDD interaction. As a control, we injected our purified Zika viral particle into an affinity column, to analyze the capacity to form unspecific interactions (Fig. 5D). We also observed in lane 1 MW marker, lane 2 envelope ZIKV protein band, and lane 3 elution band, where no band is observed, this experiment confirms that ZIKV interact specifically with the affinity column. In Fig. 5E and F we show the 3D structures of NTDyn (edited from PDB code: 6F1T) and NDD (PDB code: 5OWO) respectively for size comparison.

## Discussion

The recent SARS CoV-2 outbreak has been an example of how fast viral infections can be globally spread in a minimal fraction of time, and how extremely vulnerable to the action of virus we are. The virus has a high mutation rate, this helps the virus to cope with antivirals and to gain resistance against them. Finding host cells’ molecules that interact with the virus and targeting that interaction with new drugs could diminish the probability of antiviral resistance. In this work, we evaluate the role of human cytoplasmic dynein-1 (dynein) in the replication cycle of the Zika virus (ZIKV) and obtained clear evidence that indicates that those two proteins interact in vitro as well as in infected cells. First, we investigated whether this interaction could be present in infected cells, by infecting Vero cells, fixing samples at 12, 24, and 48 h post-infection. We revised the immunolocalization of the ZIKV and the naturally occurring dynein HC (Fig. 1), the maximum colocalization was at 24 h, this is consistent with the reported initiation of viral protein synthesis in this cell lane [17]. The colocalization of the protein-antibody complex in confocal microscopy could have interactions around 400–600 nm due to the limited resolution of the optical system. To increase the resolution of our method, we used the proximity ligation assay, which has a theoretical maximum limit of 40 nm. There are some limitations or false interpretations of this technique [18]. The loss of the interaction after 18 h, could mean that the ZIKV is being processed inside vesicles, so there is no need for a direct interaction of dynein with the virus. The kinetics seems consistent with what was observed in our colocalization experiments, and with the colocalization showed by Shrivastava et al. These results suggest that in an early stage of the infection, dynein would have the task of transporting ZIKV by interacting directly with the E protein. Also, we observed an increase in the expression of dynein HC in infected cells at 12 h (Fig. 3) On the other hand, during the transport of the virus towards the perinuclear zone, the virus is transported in the endosome. For this, the necessary machinery is GTPase Rab 5 or 7, which regulates transport to early or late endosomes, respectively [19]. These membrane proteins of the endosome interact with the cargo adaptor on one end, and on the other end, it interacts with the dynein–dynactin complex [20]. The endosome membrane proteins, and the coiled-coil cargo adaptor whose length we have calculated to be approximately 66 nm [21], would not allow us to obtain a signal in the PLA (< 40 nm). In addition, during immunoprecipitation, the RIPA buffer solubilizes the lipids from the native membranes, since ZIKV is inside the vesicle, in case the interaction that we are capturing could be with the ZIKV inside a membrane vesicle, the virus would be released of the vesicle, and it will not interact with dynein HC. Since we are still detecting dynein-ZIKV interaction with the immunoprecipitation experiments, it means that we are capturing a complex with direct interaction (Fig. 4). We have also performed kinetics of the infection, with a maximum of PLA signal at 18 h.p.i. and after this time, we observed a decrease of the PLA dots (Fig. 2). We increased the MOI from 1 to 5 in order to check some insights of direct interaction at the beginning of the replication cycle with no positive PLA at 12 h or less, nor after 24 h (data not shown). We propose that after 18 h, the viruses are being processed inside vesicles, so the direct interaction between ZIKV and dynein is not detectable. Taken together, our data led us to propose the next addition on the ZIKV replication cycle; since the ZIKV release of the first new virions is approximately 24 h post-infection, and we are losing both, colocalization and PLA signals after 18 h, we believe that ZIKV’s replication cycle step on which dynein participates should be when newly synthesized viral proteins in the cytoplasm [11]. The co-localization assay and PLA could only determine closeness and no interaction in vitro, which led us to analyze the interaction through the immunoprecipitation of the complex, this assay in infected Vero cells guarantees that this interaction occurs naturally during the viral replication cycle. We propose three possible scenarios; first, we suggest that the ZIKV polyprotein starts translating on the cytosol, where it hijacks the infection-dependent highly expressed dynein (12–18 h) that transports the full or partially non-processed polyprotein to the viral factories (not direct dynein-ZIKV interaction), where the virions will be processed, assembled and then, transported into the Golgi apparatus to its final maturation step before it is released to the extracellular space (24 h). There is evidence in the literature of a non-processed polyprotein composed of C-prM-E-NS1 proteins of the YFV flavivirus, synthesized with the in vitro translation system in rabbit reticulocyte lysates [13]. This translation system in rabbit reticulocyte lysates has no microsomal membranes. The second scenario is that the dynein-ZIKV complex could be formed due to the translocation of factors from the ER lumen to the cell surface that could facilitate in yet unknown ways apoptotic signaling cascade [8]. It has been observed that, under stress conditions, the permeability of the ER allows luminal proteins to be released or translocate to the cytoplasmic side of the ER [22]. This would also require the transport of E protein by the dynein to the viral replication factories. The third and final scenario we are proposing is a dual polyprotein topology an unknown process of post-translational translocation leading to the non-uniform topology, where there is an equilibrium of envelope protein copies in luminal or in cytosolic compartments [8]. In hepatitis B virus (HBV) has been observed that all envelope proteins synthesized in transfected cells or in a cell-free system adopt more than one transmembrane orientation [23]. In this way, vesicles with E protein from ZIKV facing cytosol could bind to dynein and the whole vesicle will be transported by the molecular motor in order to reach the viral factories. Thus, we have shown strong evidence of the first non-coiled-coil protein that interacts with dynein without a cargo adaptor or dynactin. In order to prove this strong interaction with the complete Zika virion, we attached the His6NTDyn into an affinity Nickel column and then, we bound an enriched extract of virions from infected Vero cells to this column and elute the samples. We show that NTDyn was able to bind Zika virions since both molecules co-elute with imidazole. Once we eliminate the helical-bundles 1, 2 and 3 (residues 202–504) of the NTDyn by using the NDD (Fig. 5B and F), we carry out the same experiment where we observe the same result the obtained with the NTDyn, this delimits the interaction to the first 201 amino acids of the amino-terminal fraction of the heavy chain of dynein. This could be the answer to why the ZIKV binds to dynein without dynactin or cargo adaptor; dynactin and the cargo adaptors BICDR, BICD2 and HOOK3 bind to the helical bundles 1, 2 and 3, the interaction we are proposing is in the ‘opposite face’ of dynein. Although we do not know the mechanism of this interaction and whether ZIKV binding promotes dynein processivity we suggest that it is a retrograde transport function.

**Fig. 6:**
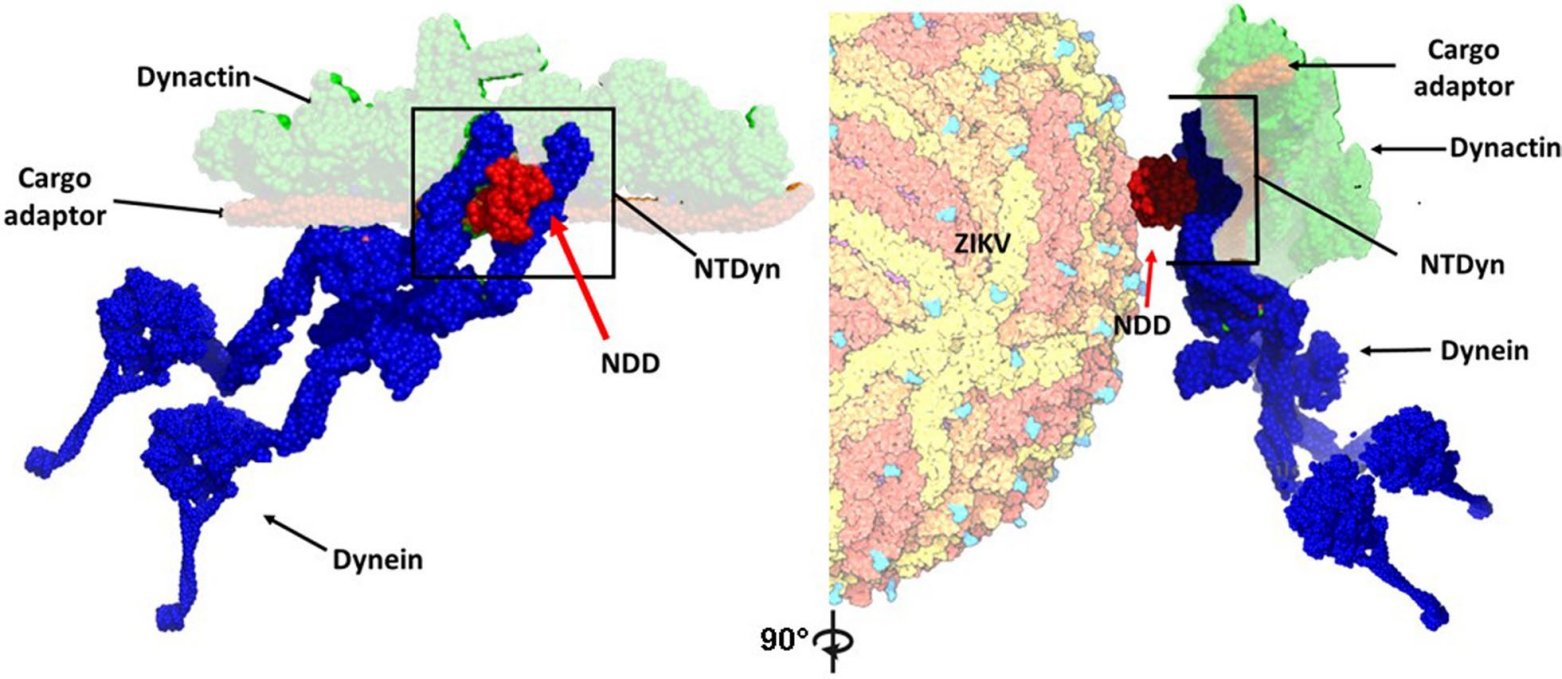
NDD is exposed to ZIKV interaction in a retrograde traffic complex. In blue, dynein forms a complex with dynactin in green, and in brown, the cargo adapter. We emphasize the position of the NDD in red within the complex and its ability to be recruited by the envelope protein. PDBs 3VKH [16] and 5NW4 [14]

In addition, the position of NDD in the complex would give it the ability to interact with the ZIKV envelope, perhaps during its retrograde movement (Fig. 6). In comparison with similar studies, such as Brault et al, where they characterize an interaction between Tctex-1 and prM, which could be attributed to a different biological role, due to the ability of Tctex-1 to lodge in different cellular compartments [24]. Shrivastava et al, attribute a retrograde trafficking role to an observed interaction between the LC8 dynein light chain and DENV pE, although does not clearly address the location of the interaction [12].

## Conclusions

We have shown strong experimental evidence that demonstrates the interaction between the E protein from the Zika virus and the intact dynein, specifically with the dimerization domain of this molecular motor, this interaction occurs during the viral replication cycle between 18 and 24 h post-infection, this interaction is an uncharacterized step in the viral replication cycle that could be targeted to design a new drug that stops virus spreading into the currently infected patients.

## Materials and methods

### Immunofluorescence ASSAY

Vero cells were seeded 1 day prior to infection at a minimum concentration of 1 × 10^5^ cells/ml in cell culture flasks. Cells were infected with ZIKV (MR766 strain) at a multiplicity of infection (MOI) of 1. Adsorption was carried out for 1 h at 4 °C to prevent virus entry and synchronize infection of cells. Cells were washed twice with a cold medium to remove unbound virus and replenished with a prewarmed medium. The coverslips were taken out of the culture at 12, 48 and 72 h post-infection (h.p.i.) under sterile conditions, washed with 0.01 M phosphate buffer saline pH 7.2 (PBS) and fixed in ice-cold acetone for 10 min at − 20 °C for experiments. The fixed cells were washed with PBS and blocked with 1 % bovine serum albumin (BSA) in PBS for 30 min. For dual staining, NTDyn was labelled with our labmade polyclonal antibody (rabbit) and the E protein using the pan-flavivirus monoclonal antibody 4G2. The primary antibodies were added simultaneously and incubated for 1 h followed by washing with PBS. This was followed by incubation with appropriate secondary antibodies conjugated to fluorescent dyes, Alexa 488 or Alexa 594 added simultaneously. DAPI (4, 6 diamino-2-phenylindole) was used to stain the nucleus in all experiments. At the end of the staining process, coverslips were washed and mounted onto slides using a mounting medium. Control samples were non-infected cells processed with the same procedures described previously and were included in all experiments as mock-infected cultures. Cells were observed and images were digitalized in a Zeiss 700 Confocal microscope.

### Envelope and dynein protein expression kinetics

Vero cells at 80% confluency were infected with ZIKV at an MOI of 5. Infection kinetics at 8, 12 and 24 h were performed. Uninfected Vero cells and Vero cells treated with a heat-inactivated virus (mock) were used as controls. Cells were then lysed with RIPA buffer (100 mM Tris-HCl pH 8.3, 2% Triton X-100, 150 mM NaCl, 0.6 M KCl, 5 mM EDTA) in the presence of one tablet of the protease inhibitor cocktail per 25 mL (Complete, Invitrogen). Cell lysates were analyzed by 12% SDS-PAGE for 80 min at 100 V (Mini-Protean Cell, Amersham Biosciences, Piscataway, NJ, USA) and transferred into nitrocellulose membranes (BIO-RAD) was carried out at 120 V for approximately 2 h). Membranes were blocked with PBS-Tween-5 % milk for 1 h and then washed four times with PBS-Tween. Membranes were then incubated overnight with the primary antibodie (lab-made for NTDyn (Additional file 1), and anti-E mAb 4G2 (Merk-Millipore)) at 4 °C. After this incubation, the membranes were washed again with 1X PBS and incubated with HRP-coupled secondary (Invitrogen) for 1 h. After another round of 4 washes with PBS-Tween, the membranes were developed in the presence of the chemiluminescence developer reagent (Thermo Scientific) following the manufacturer’s instructions, using the ChemiDoc kit (BIO-RAD) and digitalized with the ChemiDoc XRS System (BIO-RAD).

### Proximity ligation assay

Vero cells were seeded 1 day prior to infection at a minimum concentration of 1 × 10^5^  cells/mL on glass coverslips in a 24 well plate. Cells were infected with ZIKV (MR766 strain) at a MOI = 1. Then cells were fixed with formaldehyde 4% for 10 min at 8, 12, 18, 24 and 48 h.p.i. After this, the cell culture was washed with wash buffer (PBS Tween 0.1%) and permeated with PBS- Tween 0*.*2% for 10 min. Once removed the permeabilization buffer, samples were blocked with blocking buffer (5 % bovine serum albumin in PBS) for 1 h at 37 °C and continued with wash and incubation with the primary antibodies 1:100 dilution overnight at 4 °C with blocking buffer. Mock- infected cells were incubated with both primary antibodies and control infected cells were incubated without the anti-E mAb, both samples were run in parallel as negative controls. The next day, samples were washed, and the PLA probe was added in 1:5 dilution with blocking buffer for 1 h at 37 °C in a humidity chamber. The sample was washed and incubated with the ligation mix (6 µL of the concentrated ligation buffer with water for a total reaction volume of 30 µL and 1 µL of ligase) for 30 min at 37 °C. Then, the sample was washed and incubated with the amplification mix (6 µL of the concentrated amplification buffer with water for a total reaction volume of 30 µL and 0.5 µL of polymerase) for 100 min at 37 °C. After this incubation, the cells were washed with wash buffer B and the sample was then incubated with 4,6-diamidino-2-phenylindole (DAPI) to stain nuclei for 10 min under gentle agitation. Next, the samples were washed one time with B buffer 1X and one time with buffer B 0.01X for 1 min. Finally, the samples were mounted and analyzed in a confocal microscope (Zeiss 700). Results were quantified by ImageJ and expressed as several dots/cells.

### Immunoprecipitation envelope protein–dynein

Vero cells were seeded 1 day prior to infection in cell culture flasks. The cells were infected with the ZIKV MR766 strain at a MOI of 5. The medium was removed 24 h later and cross-linked with 1 % formaldehyde and incubated for 10 min at 37 °C, cross-linker was removed washing twice with cold PBS 1X. Cells were incubated with RIPA buffer for 1 h on ice, then the sample was centrifuged at 13,000 g for 20 min at 4 °C and the supernatant was recovered. 20 µL of magnetic beads were taken and washed four times with solution A (25 mM Tris HCL pH 7.5, 125 mM NaCl, 2.5 mM EDTA, 2.5 mM EGTA, 2.5 mM NaF, 0.1 70% Triton × 100). 10 µL of Antibody (Anti-E, 4G2) were added and incubated overnight at 4 °C. The next day, the supernatant of the samples was added, and the sample was incubated overnight at 4 °C. The magnetic beads were then retrieved using the magnet and washed four times with wash solution B (25 mM Tris HCL pH 7.5, 125 mM NaCl, 2.5 mM EDTA, 2.5 mM EGTA, 2.5 mM NaF, 0*.*5% Triton X100). Finally, 50 µL of sol C (25 mM Tris pH 8.0, 2.5 mM EDTA, 0*.*1% SDS) are added and incubated at 65 °C for 30 min to recover the supernatant, which was analyzed by Western Blot with the Mab Anti-E, Pab Anti-NTDyn.

### ZIKV envelope protein expression and purification

The complete *E. coli* codon-optimized coding sequence for the ZIKV envelope protein (E) (corresponding to UVE/ZIKV/1947/UG/MR766) was a generous gift from Prof. Glaucius Oliva from the University of Sao Paulo. The DNA corresponding to the E-protein without the membrane domain (Ep) was subcloned to pRSET A plasmid with an N- terminal His6-tag. This version of E protein was expressed in *E. coli* SoluBL21 strain (Genlantis) into inclusion bodies (IB). The E protein was purified [25] with the following modifications, we started the solubilization of the IB with 8 M urea 6 h at room temperature. Subsequently, the solubilized E protein was applied into a Histrap HP 5 ml pre-equilibrated with the refolding buffer (100 mM Tris pH 8.0, 400 mM L-Arg HCl, 2 mM EDTA, 5 mM reduced glutathione, 0.5 mM oxidized glutathione, and 5 % glycerol) and then eluted with the refolding buffer with 500 mM imidazole. The His6-tag was cleaved with the TEV protease and concentrated by centrifugation (1650 g at 4 °C) in an Amicon Ultra 15 concentrator with 30 kDa cutoff. 500 µL of the concentrated E protein, was purified further with a Superdex 200 10/300 Increase gel filtration column (GE Healthcare). Fractions containing the E protein were pooled and concentrated at 10 mg/ml.

### Expression and purification of the N-term dynein heavy chain and dimerization domain

Plasmids for *E. coli* codon optimized NTDyn and NDD were a generous gift from Prof. Andrew Carter from the Laboratory of Molecular Biology of the Medical Research Council, Cambridge, UK. Both proteins were individually purified using the same protocol. The *E. coli* codon-optimized human dynein heavy chain N-Terminus residues 1–560 (NTDyn) and human cytoplasmic dynein dimerization domain 1–201 (NDD) with N-terminal His6-tag and TEV protease cleavage site into the pRSET (A) plasmid was expressed in *E. coli* SoluBL21 strain as described previously with some modifications [15]. Once expressed, the cells were sonicated (500W Ultrasonic processor Cold Palmer CV334), with 5–35 s pulse-rest cycles for 3 min at 50% power with buffer A (30 mM Tris pH 7.5, 200 mM NaCl, 2 mM MgCl_2_, 25  mM imidazole, 1 mM benzamidine, 1 mM PMSF, 1 mM beta-mercaptoethanol). Next, the cell lysate was ultra-centrifugated at 165,000×*g* for 30 min at 4 °C; the clarified material was filtered with a 0.22 µm membrane and then applied a HisTrap HP 5 ml (GE Healthcare) and eluted with buffer B (30 mM Tris pH 7.5, 200 mM NaCl, 2 mM MgCl_2_, 500 mM imidazole, 1 mM benzamidine, 1 mM PMSF, 1 mM beta-mercaptoethanol). The eluted protein was desalted with a desalting column (HiPrep 26/10 GE) and digested for 18 h at 4 °C with the TEV protease. The removed His6-tag and the TEV protease were bound to the HisTrap affinity column, and the protein was eluted in the washing step. The fractions were concentrated to 500 µL by Amicon Ultra 15 concentrators with 30 kDa cut-off and applied to a Superdex 200 10/300 Increase gel filtration column (GE Healthcare). Individual fractions containing the NTDyn and NDD were pooled and concentrated at 10 mg/mL (Additional file 1, Fig. 8).

### NTDyn or NDD-ZIKV interaction on IMAC

For experiments A and B 100 µg of protein His6-NTDyn or His6-NDD was bound to 1 mL HisTrap HP previously equilibrated with phosphate-buffered saline (PBS 1X) pH 7.4 and then washed with 5 mL of the same buffer to later bound the viral particle in Molar ratio 10:1 protein: virus. Viral particles were harvested 24 h p.i., purified and injected through the column and subsequently eluted with 5 mL of elution buffer (30 mM Tris pH 7.5, 200 mM NaCl, 2 mM MgCl_2_, 500 mM imidazole, 1 mM benzamidine, 1 mM PMSF, 1 mM beta-mercaptoethanol). For the protein:antibody complexes, 100 µg of NTDyn or NDD interacted with anti-NTDyn or anti-NDD polyclonal rabbit antibodies for 30 min in a 1:4 protein: antibody ratio. Once the complexes were formed, they were bound to the affinity column previously equilibrated with (PBS) pH 7.4 and washed with 5 mL of the same buffer to later bound the viral particle in Molar ratio 10:1 protein: virus. Viral particles were harvested 24 h.p.i., purified and injected through the column and subsequently eluted with 5 ml of elution buffer (30 mM Tris pH 7.5, 200 mM NaCl, 2 mM MgCl_2_, 500 mM imidazole, 1 mM benzamidine, 1 mM PMSF, 1 mM beta- mercaptoethanol). After elution, fractions were analyzed by SDS-PAGE (Additional file 1).

## Supplementary Information

**Additional file 1**. Supplementary material.

## Data Availability

Not applicable.
