# Supplementary material for: Interaction of the Zika virus with the cytoplasmic dynein-1
Source: Virol J. 2023 Mar 6;20:43. doi: 10.1186/s12985-023-01992-6 (PMC9987375; doi:10.1186/s12985-023-01992-6)
Supplement: Supplementary file 1 — Additional file 1. Supplementary material. [file 12985_2023_1992_MOESM1_ESM.docx]

## Supplementary material

- 1. **Anti-NTDyn and anti-NDD pAbs production.** Both antibodies were individually produced and used the same protocol. Four 10-12 weeks-old Rabbit NZB Crl:KBL(NZW)BR were obtained from the breeding facilities at the CINVESTAV (Centro de Investigation y de Estudios Avanzados del Instituto Politecnico Nacional). All animals were housed and handled in accor- dance with institutional guidelines. The rabbits were immunized with three doses of 100 µg of NTDyn or NDD recombinant proteins. These immunizations were administered subcutaneously with complete Freund’s adjuvant at the first inoculation, and with the incomplete Freund’s adjuvant, at the first, third and fifth week. In the seventh week, the rabbits were sacrificed and the antibodies in the serum were purified by protein A-Sepharose 4B (Invitrogen).
  2. **Far-Western blot NTDyn-E protein.** To address the interaction between the recombinant E protein and NTDyn we did a far-western blot. Initially, we transferred in five lanes of a PVDF membrane 100 µg of the recombinant E protein in each lane. After membrane blockade we made it interact with different amounts of the recombinant NTDyn. As a result, we observed in figure 8 an increase in the intensity of the signal that corresponds to the increase in the concentration of NTDyn. As a control we used a lane transferred with 100 µg of NTDyn to which we did not interact with anything, this allowed us to observe the intensity of the signal generated by the reactivity of the antibody, for the negative control we took a lane transferred with recombinant E protein and made it interact with 100 µg of BSA. As a result, we do not observe a signal, which shows us the specificity of the interaction. These results corroborated our hypothesis and led to the analysis of this interaction between recombinant proteins. Methodology. Five lines of the recombinant purified E protein and one of NTDyn were analyzed by 12 percent SDS-PAGE for 80 min at 100 V (Mini-Protean Cell Amersham Biosciences, NJ, USA) and transferred into PVDF membranes (BIO-RAD) was carried out at 120 V for approximately 2 hours). Membranes were blocked with PBS-Tween-5 percent milk for one hour and then washed four times with PBS-Tween. Membranes were then incubated two hours with 0, 10, 100, 1000 g 120 min at TA with PBS. Then the membranes were incubated overnight with the primary antibodies at 4 °C. After this incubation, the membranes were washed again with 1X PBS and incubated with HRP-coupled secondary (Invitrogen) for one hour. After another round of 4 washes with PBS-Tween, the membranes were developed in the presence of the chemiluminescence developer reagent (Amersham ECL Western Blotting Detection Reagent Cytiva) and digitalized with Amersham Imager 600 System GE Healthcare.


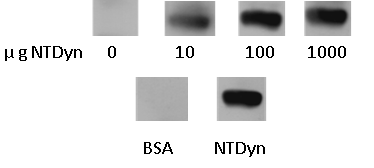


**Fig. 7.** Immunoblot depicting far western Blot E protein-NTDyn. In the four upper lanes the interaction of the E protein with different concentrations of NTDyn is shown, in two lower lanes the controls of NTDyn and interaction with BSA are shown.

- 1. **NTDyn and NDD gel filtration chromatography.** The NTDyn fragment and the NDD were both purified following the protocol referred in material and methods. Using an affinity chromatography step and a gel filtration step. Finally, the fractions resulting from gel filtration were concentrated and analyzed with SDS-PAGE.


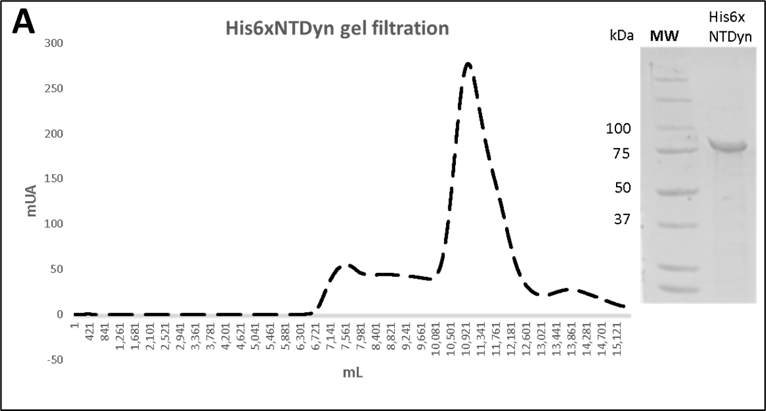

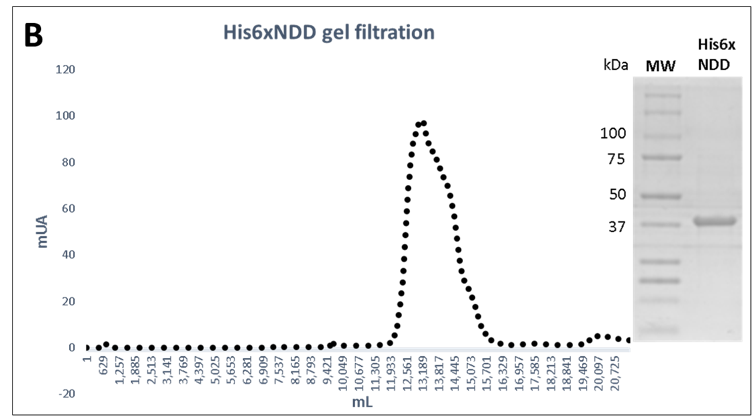


**Fig. 8.** NTDyn and NDD purification. A. NTDyn gel filtration chromatography and Coomassie-stained PAGE-SDS gel, B. NDD gel filtration chromatography and Coomassie-stained PAGE-SDS gel

C NTDyn and NDD gel filtration chromatography

**NTDyn+AbNDD**

**NTDyn+AbNTDyn**

**Fig. 9.** NTDyn-antibodies complex A. size-exclusion chromatogram, of the NTDyn-anti-NTDyn and NTDyn-anti-NDD complexes on the right side the Coomassie-stained PAGE-SDS gel of the corresponding elution peaks, is shown.


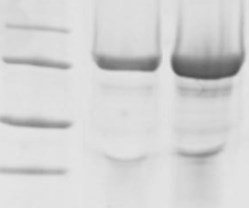

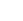

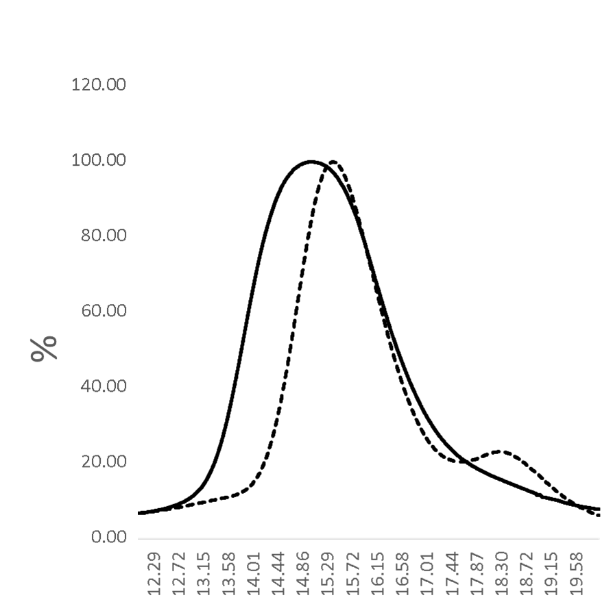

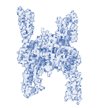

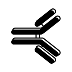

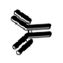

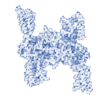

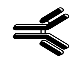

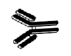

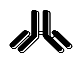

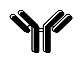


**NTDyn+AbNDD**

**NTDyn+AbNTDyn**

**%**

**100 kDa**

**75 kDa**

**NTDyn**

**50 kDa**

**mL**

**25 kDa**

**IgG**

**D.NTDyn antibodies complex size-exclusion chromatography profiles.** To verify that the anti-NDD antibody was bind- ing only to the NDD of the NTDyn fragment, we analyzed this com- plex by gel filtration and compared it with the NTDyn-anti- NTDyn complex. In this way, we were able to analyze the interaction capacity of the antibody since both polyclonal antibodies have different binding sites hence the NTDyn-anti-NTDyn complex would show a reduction in its elution volume compared to the NTDyn-anti-NDD complex. Because the NTDyn-anti-NDD complex -NDD would not have antibodies bound to the alpha-helices of the NTDyn fragment. Which would give us the ability to test the interaction be- tween the alpha-helices of NTDyn and ZIKV. As a result, we observe in figure 6A, right panel, the chromatogram corresponding to the Size-exclusion chromatography of both complexes, where it is shown that the maximum elution peak of the NTDyn-anti-NDD complex occurred at 15.02 ml. While the maximum peak of the NTDyn-anti-NTDyn complex occurred at 15.39 ml, this result is consistent with expected, since the NTDyn-anti-NDD complex should have fewer bound antibodies, so it would show a lower elution vol- ume. The chromatography fractions were analyzed by SDS-PAGE, where the presence of bands corresponding to the NT-Dyn fragment and IgG was observed.Methodology. Two different experiments were performed, the first was the injection of the NTDyn and anti-NTDyn polyclonal antibody complex, this was injected after a 30 mins incubation in a 1:4 molar ratio NT- Dyn:antibody to allow interaction. The second experiment consisted of injecting the NTDyn and anti-NDD antibody complex. These experiments were designed to block the NTDyn against the interaction with the ZIKV, using the Anti-NTDyn pAb, and by using the NDD pAb, we are blocking only the NDD but the helical bundles 1, 2 and 3 are still free for the interaction. Experiments were performed using a Superdex 200 10/300 size exclusion column (GE Healthcare) previously equilibrated with PBS 1X pH 7.4 buffer at room temperature at a flow rate of 0.5 ml/min. The resulting fractions were analyzed with respect to output volume and by SDS-PAGE.
